# The Inhibition Effects of Shenmai Injection on Acetylcholine-Induced Catecholamine Synthesis and Secretion by Modulating Nicotinic Acetylcholine Receptor Ion Channels in Cultured Bovine Adrenal Medullary Cells

**DOI:** 10.1155/2020/8514926

**Published:** 2020-12-15

**Authors:** Xiting Zhang, Lin Li, Yi Wang, Haoping Mao, Lijuan Chai, Lin Miao, Shuang Wang, Xiumei Gao, Han Zhang

**Affiliations:** ^1^Institute of Traditional Chinese Medicine, Tianjin University of Traditional Chinese Medicine, Tianjin 301617, China; ^2^State Key Laboratory of Component-Based Chinese Medicine, Tianjin University of Traditional Chinese Medicine, Tianjin 301617, China; ^3^Key Laboratory of Pharmacology of Traditional Chinese Medical Formulae, Tianjin University of Traditional Chinese Medicine, Ministry of Education, Tianjin 301617, China; ^4^Laboratory of Pharmacology of TCM Formulae Co-Constructed by the Province-Ministry, Tianjin University of Traditional Chinese Medicine, Tianjin 301617, China

## Abstract

Shenmai injection (SMI) has been widely used for the treatment of cardiovascular diseases in China. Cardiovascular disorders are often related to excessive catecholamine (CA) secretion. Here, we report the effects of SMI on CA secretion and synthesis in cultured bovine adrenal medullary cells. We found that SMI significantly reduced CA secretion induced by 300 *μ*M acetylcholine (ACh). Cotreatment with SMI (10 *μ*L/mL) and either of the ACh receptor *α*-subunit inhibitors, HEX (*α*3) or Dh*β*E (*α*4*β*2), did not produce any further inhibition, indicating that SMI may play a role through *α*3 and *α*4*β*2 channels. Furthermore, SMI reduced tyrosine hydroxylase (TH) activity induced by ACh by inhibiting the phosphorylation of TH at Ser19 and Ser40. TH is phosphorylated at Ser19 by Ca^2+^/calmodulin-dependent protein kinase II (CaM kinase II) and at Ser40 by protein kinase *A* (PKA). KN-93 and H89, the antagonists of CaM kinase II and PKA, respectively, inhibited the ACh-induced phosphorylation at Ser19 and Ser40, and the addition of SMI did not augment the inhibitory effect. Taken together, our results show that SMI likely inhibits CA secretion by blocking TH activity at its Ser19 and Ser40 sites.

## 1. Introduction

Catecholamines (CAs) are a class of neurotransmitters including norepinephrine (NE), epinephrine (E), and dopamine (DA). CAs regulate normal cardiovascular functions at physiological levels, while excessive secretion can aggravate cardiovascular diseases such as heart failure, atherosclerosis, coronary heart disease, hypertension, and so on [1].

Bovine adrenal chromaffin cells, also called adrenal medullary cells, were derived from embryonic neural crests with homologous function in sympathetic neurons [2]. Hence, bovine adrenal medullary cells were widely used to detect the effect of various medicines or chemicals on catecholamine secretion and synthesis. Researchers found that there are at least three distinct types of ionic channels that participate in catecholamine secretion, including nicotinic acetylcholine receptor (nAChR) ion channels, voltage-dependent Na^+^ channels, and voltage-dependent Ca^2+^ channels [3]. In cultured bovine adrenal medullary cells, Ach and veratridine were reported to be activators of the nicotinic acetylcholine receptor and voltage-dependent Na^+^ channels, respectively. Both ACh and veratridine caused Na^+^ influx and depolarization of membrane after they acted with the above receptors [4]. Then, depolarization of membrane could cause the opening of voltage-dependent Ca^2+^ channels. Besides, high K^+^ (HK) directly activated voltage-dependent Ca^2+^ channels to increase Ca^2+^ influx without increasing Na^+^ influx [5]. Ca^2+^ influx is the essential trigger for CA synthesis and secretion [6]. It was found that the final raise of introcellular (Ca^2+^) induced by ACh, Ver, and HK induced the secretion of catecholamine. On the other hand, it was known that biosynthesis of CA began with dihydroxyphenylalanine (DOPA) which was converted to dopamine (DA); the latter was then modified into norepinephrine (NE) and epinephrine (E) [7]. Tyrosine hydroxylase (TH), which catalyzed the rate-limiting step in catecholamine biosynthesis [8], was activated via phosphorylation at the Ser19, Ser31, and Ser40 sites in correspondence to following multiple protein kinases, including Ca^2+^/calmodulin-dependent protein kinase II (CaM kinase II), extracellular signal-regulated protein kinase (ERK), and cAMP-dependent protein kinase A (PKA) [9, 10].

Shenmai injection (SMI), based on the traditional Chinese prescriptions of Shenmai San that was recorded in the 1186 AD “Yixue qiyuan” account of Zhang Yuansu, was approved by China Food and Drug Administration (CFDA) for the treatment of heart failure in 1995 [11]. Consisting of aqueous extracts from *Panax ginseng* C.A. Mey (Renshen) and *Ophiopogon japonicus* (Thunb.) Ker Gawl. (Maidong), its chemical fingerprinting has been established by high-performance liquid chromatography (HPLC) [12], and the main pharmacological active components are ginsenoside Rg1, ginsenoside Rg2, ginsenoside Rg3, ginsenoside Re, ginsenoside Rb1, and ophiopogonin *D* [13, 14]. It has been widely used for hypertension, coronary heart diseases, stroke, chronic pulmonary heart disease, viral myocarditis, heart and respiratory failure, cerebral infarction, and malignant diseases [15–17].

Although toxicity studies have confirmed the general safety of SMI [18, 19], its effects and mechanisms on catecholamine secretion and synthesis have not been fully investigated. Therefore, in the current study, bovine adrenal medullary cells were used to investigate the effect and the potential mechanisms of SMI on CA synthesis and secretion. First, we primary cultured the bovine adrenal medullary cells and established the ACh, Ver, and HK-induced CA secretion model to investigate the effect of SMI on CA secretion by using HPLC coupled with the electrochemical detection (HPLC-ECD) method and introcellular Ca^2+^ by an inverted fluorescent microscope. Then, we used the inhibitors of corresponding subunits of nAChR to confirm in which subunits of the channels did SMI exerts its effects on inhibiting Ca^2+^ influx and CA secretion. Finally, we detected the effects of SMI on the activity of TH and its possible phosphorylation sites to investigate the effect and mechanism on CA synthesis.

## 2. Materials and Methods

### 2.1. Materials

SMI was purchased from CTQ Pharmaceutical Group Co., Ltd. (Hangzhou, China) according to the guidelines of Good Manufacturing Practice and Good Laboratory Practice. Oxygenated Krebs-Ringer phosphate (KRP) buffer was prepared using 154 mM NaCl, 5.6 mM KCl, 1.1 mM MgSO_4_, 2.2 mM CaCl_2_, 0.85 mM NaH_2_PO_4_, and 2.15 mM Na_2_HPO_4_ with pH adjusted to 7.4. Eagle's minimum essential medium (Eagle's MEM) was purchased from Coring (New York, USA), and fetal calf serum (FCS) was sourced from Australia. Methyllycaconitine citrate (MLA) and dihydro-*β*-erythroidine hydrobromide (DH*β*E) were obtained from Merck Millipore (Massachusetts, USA) and RD (San Francisco, USA), respectively. Hexamethonium bromide (HEX), KN-93, and H89 were purchased from MCE (Shanghai, China). Antibodies against phosphorylated TH were obtained from Jianshu, China (p-THSer19 and p-THSer40), and Cell Signaling Technology (Boston, USA) (p-THSer31 and *β*-actin). The Pierce BCA Protein Assay Kit was purchased from Thermo (Waltham, MA, USA). Acetylcholine chloride (ACh), veratridine, and potassium chloride were obtained from Sigma (St. Louis, MO, USA). The cell culture reagents trypsin inhibitor, collagenase *L*, and penicillin-streptomycin and collagenase type 1 were purchased, respectively, from Nacalai Tesque (Kyoto, Japan), Wako (Kyushu, Japan), and Gibco (California, CA, USA). Finally, the TH activity detection kit was purchased from Genmed (Shanghai, China) and Fluo4-AM from Dojindo (Kyushu, Japan).

### 2.2. Isolation and Primary Culture of Bovine Adrenal Medullary Cells

Bovine adrenal glands were obtained from the City Slaughter House. The chromaffin cells were isolated from the adrenal glands by collagenase digestion as described previously [20]. The cells were cultured at a density of 5 × 10^5^ cells/well in Eagle's MEM supplemented with 10% FCS, 50 U/mL penicillin, and 50 g/mL amphotericin *B* under a humidified atmosphere of 5% CO_2_ and 95% air at 37°C in 12-well, 24-well, and 48-well plates. Primary cells harvested between 2 and 5 days of culture were used for experiments.

### 2.3. Catecholamine Secretion from Cultured Bovine Adrenal Medullary Cells

To detect the effect of SMI on CA secretion, the level of CAs in culture medium was detected by the high-performance liquid chromatographic system coupled with an electrochemical detector (HPLC-ECD). Briefly, bovine adrenal medullary cells were buffered with oxygenated KRP and washed 3 times before experiments. After incubation with the indicated agents at 37°C for 10 min, the medium was transferred immediately to a test tube containing perchloric acid (PCA) (final concentration, 0.4 M), and NE and *E* secreted into the medium were detected by HPLC-ECD. The chromatographic separation system included a RP-C18 analytical column (5 *μ*m, *φ*4.6 mm × 250 mm) and an isocratic solvent system. The flow phase (1 L) contained 100 mL acetonitrile, 100 *μ*L triethylamine, 13.6 g KH_2_PO_4_, 2.5 g octane-sulfonic acid sodium, and 0.036 g EDTA, pH adjusted to 3 with H_3_PO_4_. The flow rate was 0.6 mL/min, column temperature was 30°C, and sample injection volume was 10 *μ*L. An electrochemical detector with two channels (0 mV and +300 mV) was used to detect the signals.

Chromaffin cells were buffered with Ca^2+^ KRP and washed 3 times before experiments. After 10 min preincubation with different dilutions of SMI, cells were incubated for an additional 10 min in the presence or absence of various secretagogues. The incubation medium was then transferred immediately to a test tube containing perchloric acid (PCA) (final concentration, 0.4 M). NE and *E* secreted into the medium were directly injected and detected by HPLC-ECD as described above.

### 2.4. Measurement of Intracellular Ca^2+^ Mobilization

To examine the effects of SMI on intracellular Ca^2+^ concentration induced by 300 *μ*M ACh, cells were seeded into black-walled clear-base 96-well plates (CORING, USA) in culture media and cultured for approximately 24 h in a 37°C CO_2_ incubator. The culture media was removed, and the cells were washed with KRP buffer three times. The cells were then incubated with 2 *μ*M Fluo4-AM for 20 min at 37°C and then washed three times with Ca^2+^ (+) KRP buffer. After a final incubation in KRP buffer for 20 min at 37°C, changes in fluorescence induced by the Fluo4 complex was measured at an excitation wavelength of 494 nm and an emission wavelength of 516 nm before and after the addition of SMI (0.25–1 *μ*L/mL), with a microplate reader (SpectraMax 190, Molecular Devices, USA), and the cells were observed by an inverted fluorescent microscope (DM750 M, LEICA, Germany) at 20x amplified.

### 2.5. In Situ Detection of TH Activity

The cells cultured at a density of approximately 1 × 10^7^ cells per well were washed three times with PBS buffer. Then, the cells were incubated with the TH activity detection kit according to the manuscript's instructions. The TH activity was measured in terms of fluorescence (ex = 280 nm, em = 320 nm) by a microplate reader.

### 2.6. In Situ Phosphorylation of TH

The effect of SMI on the inhibition of phospho-Ser19, phospho-Ser31, and phospho-Ser40 of TH was assayed by SDS-polyacrylamide gel electrophoresis (SDS-PAGE) and Western blot analysis. Cells (2 × 10^6^/dish) were incubated with or without SMI (0.25–1 *μ*L/mL) for 20 min in KRP buffer. The cells were washed twice with ice-cold KRP buffer, lysed in lysis buffer, and the lysates were centrifuged at 15,000x g for 30 min.

Protein concentration was measured using an enhanced BCA Protein Assay Kit, and equal amounts of protein were mixed with 5x SDS-PAGE sample loading buffer. Following heating at 95°C for 5 min, proteins were subjected to 10% Tris-glycine gel SDS-PAGE and transferred electrophoretically to polyvinylidene difluoride membranes (BioRad). The membranes were incubated with the primary antibodies (1 : 1000) overnight at 4°C and then washed with phosphate-buffered saline containing 0.1% Tween 20 (PBS-T), followed by incubation with the rabbit radish-peroxidase-labeled secondary antibody (1 : 10000) at room temperature for 1 h. The protein bands were visualized using enhanced chemiluminescence by Western blot detection reagents, and the band densities were measured by Quantity One software using the Versa Doc imaging system (BioRad). Data were expressed as the ratio of phosphorylated proteins to total proteins.

### 2.7. Statistical Analysis

All experiments were performed in triplicates, and each experiment was repeated at least 3 times. The data were presented as mean ± SEM. Statistical significance was evaluated using the *t*-test or one-way analysis of variance (ANOVA), and *p* < 0.05 was considered significant. All statistical analyses were performed using SPSS 11.5 software (SPSS Inc., Chicago, IL, USA).

## 3. Results

### 3.1. Effect of SMI on Basal and Secretagogue-Induced CA Secretion in Cultured Bovine Adrenal Medullary Cells

**Figure 1:**
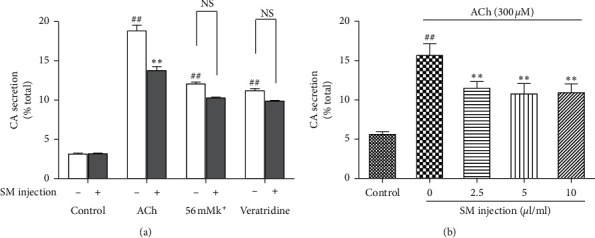
The effects of SMI on CA secretion in bovine adrenal medullary cells. (a) Effects of 2.5 *μ*L/mL SMI on basal CA secretion and on various secretagogue agonists induced CA secretion. (b) Effects of SMI on CA secretion induced by 300 *μ*M. Data are expressed in mean ± SEM based on three independent experiments each performed in triplicates (*n* = 6 per group). ^*∗∗*^*p* < 0.01 compared with ACh alone. ^##^*p* < 0.01 compared with untreated control.

To study the effect of SMI on CA secretion, bovine adrenal medullary cells were treated with various secretagogues in the presence or absence of SMI (2.5 *μ*L/mL). SMI did not affect the basal CA secretion in the control group that treated with no secretagogue (*p* > 0.05). Upon cotreatment with different secretagogues for 10 min, 2.5 *μ*g/mL SMI significantly inhibited CA secretion induced by 300 *μ*M ACh (*p* < 0.05) and caused a slight decline in 56 mM K^+^ and 100 mΜ Ver-induced secretion (*p* > 0.05) (Figure 1(a)). We also examined the effect of SMI on CA secretion induced by 300 *μ*M ACh. Cells treated with 2.5, 5, and 10 *μ*L/mL SMI for 10 min showed significantly reduced ACh-induced secretion of CA (*p* < 0.05 and *p* < 0.01) compared to Ach-treated cells only (Figure 1(b)).

### 3.2. Inhibitory Role of SMI on Catecholamine Secretion Induced by 300 *μ*M ACh

The nAChR ion channels consist of three important *α*-subunit sites—*α*3, *α*4*β*2, and *α*7. To examine the involvement of voltage-dependent nAChR ion channels on the SMI effect, we treated the cells with specific inhibitors against the *α*-subunit, namely, HEX (*α*3), Dh*β*E (*α*4*β*2), and MLA (*α*7).

**Figure 2:**
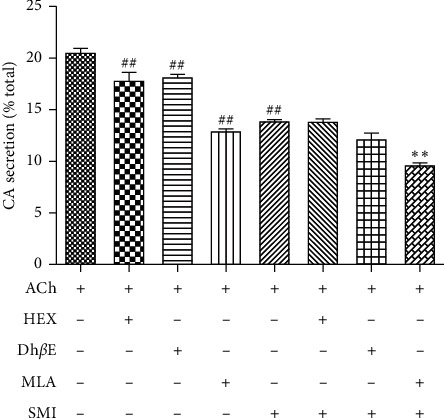
Effects of combinations of SMI and several inhibitors of *α*-subunit sites in the nAChR ion channels on catecholamine secretion induced by 300 *μ*M ACh. Data are expressed as mean ± SEM based on three independent experiments each performed in triplicates (*n* = 6 per group). ^##^*p* < 0.01, compared with 300 *μ*M ACh alone and ^*∗∗*^*p* < 0.01, compared with 300 *μ*M ACh plus SMI.

After 10 min pretreatment with (or without) SMI (10 *μ*L/mL), cells were cotreated with 300 *μ*M ACh and HEX (10 *μ*M), Dh*β*E (10 *μ*M), or MLA (10 *μ*M) for 10 min at 37°C; suitable untreated controls were included. As shown in Figure 2, HEX, Dh*β*E, and MLA each inhibited ACh-induced CA secretion (*p* < 0.05). The inhibitory effect of MLA was further augmented in the presence of SMI (*p* < 0.05), while HEX and DH*β*E did not further increase the inhibitory effects of SMI.

### 3.3. Inhibition of SMI upon Increased Ca^2+^ Influx Induced by 300 *μ*M Ach in Bovine Adrenal Medullary Cells

**Figure 3:**
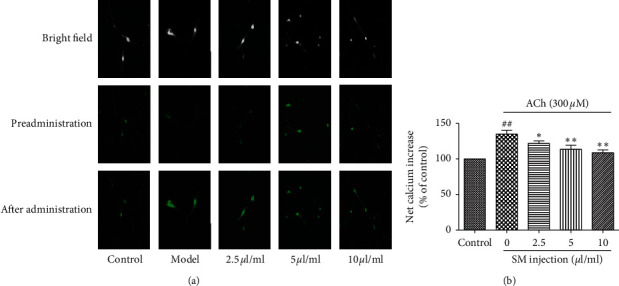
Effects of different concentrations of SMI on ACh-induced Ca^2+^ influx in cultured bovine adrenal medullary cells. (a) The cells were observed by an inverted fluorescent microscope at 200x amplified. (b) The fluorescence intensity was by detected by a microplate reader. Data are expressed as mean ± SEM based on three independent experiments each performed in triplicates (*n* = 6 per group). ^*∗*^*p* < 0.05, ^*∗∗*^*p* < 0.01 compared with ACh alone. ^##^*p* < 0.01 compared with control.

In order to further validate the inhibitory effect of SMI on nAChR ion channels, different concentrations of SMI (2.5, 5, and 10 *μ*L/mL) were used to increase Ca^2+^ influx induced by 300 *μ*M ACh, and the intracellular Ca^2+^ concentrations were measured. SMI significantly suppressed the ACh-induced Ca^2+^ influx, which is consistent with the results of CA secretion (Figure 3).

### 3.4. Effects of SMI on ACh-Induced Activity of Tyrosine Hydroxylase

**Figure 4:**
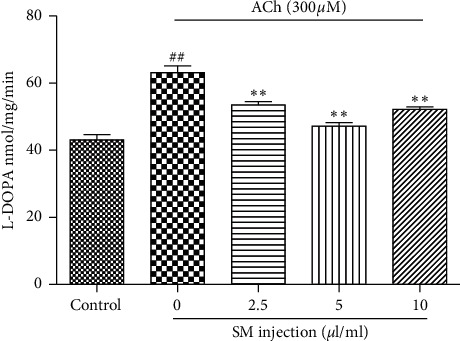
Effect of various concentrations of SMI on ACh-induced activity of TH. Data are expressed as mean ± SEM based on three independent experiments each performed in triplicates (*n* = 6 per group). ^*∗∗*^*p* < 0.01 compared with ACh alone. ^##^*p* < 0.01 compared with control.

We next examined the effect of SMI treatment on ACh-induced TH activity. Cells treated with varying concentrations of SMI (2.5, 5, and 10 *μ*L/mL) for 10 min at 37°C significantly reduced TH activity induced by ACh (*p* < 0.01) (Figure 4).

### 3.5. The Influence of SMI on TH Phosphorylation

**Figure 5:**
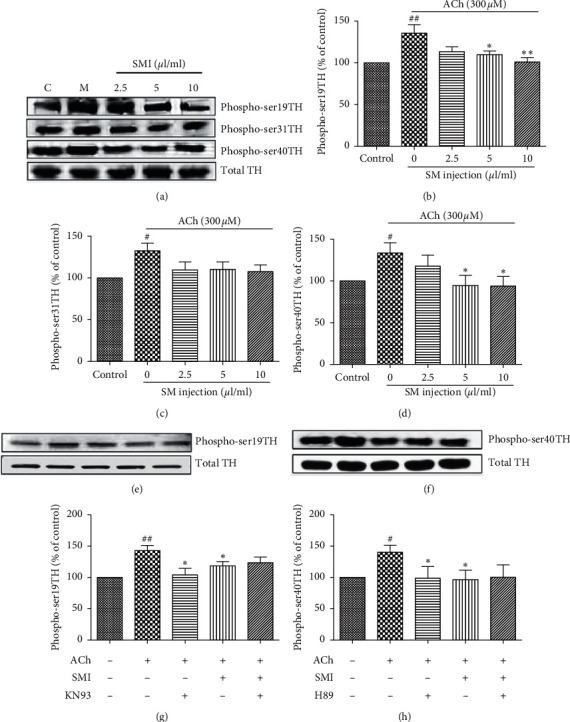
Effect of SMI on TH phosphorylation in bovine adrenal medullary cells. Phosphorylation at the Ser19, Ser31, and Ser40 sites of TH was determined and expressed as % of control TH band. (a) Effect of SMI on TH phosphorylation at Ser19, Ser31, and Ser40. (b) Effect of SMI on the ratio of the density of TH Ser19 phosphorylation induced by 300 *μ*M ACh. (c) Effect of SMI on the ratio of the density of TH Ser31 phosphorylation induced by 300 *μ*M ACh. (d) Effect of SMI on the ratio of the density of TH Ser40 phosphorylation induced by 300 *μ*M ACh. ((e), (g)) Effect of SMI and CaM kinase II on phosphorylation of TH Ser19 induced by 300 *μ*M ACh. ((f), (h)) Effect of SMI and PKA on phosphorylation of TH Ser40 induced by 300 *μ*M ACh. Data are expressed as mean ± SEM based on three independent experiments each performed in triplicates. ^*∗*^*p* < 0.05 and ^*∗∗*^*p* < 0.01 compared with ACh alone. ^#^*p* < 0.05 and ^##^*p* < 0.01 compared with control.

To determine which phosphorylated site(s) are affected by SMI, the effect of SMI on TH phosphorylation at the Ser19, Ser31, and Ser40 sites was examined. As shown in Figures 5(b) and 5(d), cells incubated with SMI (2.5, 5, and 10 *μ*L/mL) for 20 min at 37°C showed a significant decrease in phosphorylation at Ser19 and Ser40 (*p* < 0.05, *p* < 0.01), but not at Ser31 (Figure 5(c)). TH is phosphorylated at Ser19 by CaM kinase II and at Ser40 by PKA. To determine the possible mechanism of the antiphosphorylation effect of SMI, cells were treated with 10 *μ*M KN-93, a CaM kinase II antagonist, and 10 *μ*M H89, a PKA antagonist. While both antagonists significantly suppressed the stimulatory effect of 300 *μ*M ACh on TH activity (*p* < 0.05), addition of SMI (10 *μ*L/mL) did not augment the inhibitory effect of either KN-93 or H89 (Figures 5(g) and 5(h)).

## 4. Discussion

In the present study, we found that SMI reduced the CA secretion induced by ACh in adrenal medullary cells, while it had no effect on veratridine and HK-induced catecholamine secretion. We also found that all the three doses of SMI (2.5 *μ*L/mL, 5 *μ*L/mL, and 10 *μ*L/mL) inhibited ACh-induced Ca^2+^ influx, a prerequisite for the secretion and synthesis of CAs, which was consistent with the results of CA secretion. To the best of our knowledge, this is the first time that SMI mediated inhibition of the function of nAChR ion channels and CA secretion in cultured bovine adrenal medullary cells.

Nicotinic ACh receptor (nAChR), as an ionotropic receptor, was widely reported to be involved in catecholamine secretion and synthesis [21]. Because of its important role in regulation of the nerve system and involvement in a variety of diseases, nAChR was well investigated for decades from its structure to the physical functions. It was reported that *α*3, *α*4*β*2, and *α*7 as nAChR ion channels important *α*-subunit sites play an important role on CA secretion. The most relevant nAChR for CA secretion is the heteromeric one composed of *α*3 and *β*4 subunits [22]. The *α*4*β*2 nAChR X-ray structure, recently elucidated, because of its heteromeric composition deserves further discussion [23]. A typical *α*7 agonist like choline was shown to stimulate nAChRs on chromaffin cells to induce catecholamine secretion [24]. Ginsenoside Rg3, a chemical from *Panax ginseng*, was found to be voltage-dependent to inhibit ACh-induced peak inward currents by *α*3*β*2, *α*3*β*4, *α*4*β*2, and *α*4*β*4 subunits. However, the same Rg3 effect on *α*7 nAChR channel activity was not observed [25]. It was reported that ginsenoside Rg2, a chemical in SMI, had been shown to block the nicotinic acetylcholine receptors in bovine chromaffin cells by affecting the acetylcholine- (Ach-) induced currents in heteromeric receptors *α*3*β*4, *α*3*β*2, *α*4*β*4, and *α*4*β*2, without affecting the acetylcholine- (Ach-) induced currents in *α*7 human receptors [26]. In the present study, we also found that cotreatment with SMI and the AChR *α*7 subunit antagonist MLA significantly augmented the inhibition of ACh-induced CA secretion compared to SMI treatment alone, whereas the combination of SMI with *α*3 and *α*4*β*2 antagonists HEX and Dh*β*E did not produce any further inhibition. These data indicated that SMI attenuates ACh-induced CA secretion potentially by preferentially inhibiting *α*3 and *α*4*β*2 channels with no effect on *α*7 channel. These results were consistent with the findings in the previous reports on ginsenosides Rg2 and Rg3.

Except for the catecholamine secretion, chromaffin cells are also an important method for the catecholamine synthesis study [27]. In our present study, we found that SMI significantly stimulated ACh-induced catecholamine synthesis indicating a possible mechanistic basis of reducing CA secretion. Then, the mechanisms were also detected by testing the effect of SMI on tyrosine hydroxylase (TH) activity. In bovine adrenal medullary cells, TH catalyzes the conversion of tyrosine to *L*-3,4-dihydroxyphenylalanine (DOPA), the rate-limiting step in CA biosynthesis. Therefore, TH activity is essential for the synthesis of catecholamine. In the present study, SMI inhibited the ACh-induced TH activity. It was reported that TH was regulated by an allosteric activation-induced short-term regulation and an enzyme induction-induced long-term regulation. TH activity is acutely regulated by various modifications such as phosphorylation [28]. Phosphorylation at Ser40 in TH was essential to active the enzyme, while Ser19 phosphorylation had no direct effect on TH activity, but it increases TH activity indirectly by increasing the rate of phosphorylation at Ser40 [29]. So, clarifying the effect of SMI on phosphorylation of TH was important to demonstrate the stimulation of catecholamine synthesis and the TH activity. We found that SMI reduced the levels of phopho-Ser19 following ACh induction, which may likely decrease the phosphorylation rate of Ser40 and eventually decrease TH activity. In this study, KN-93 (an inhibitor of CaM kinase II) and H89 (an inhibitor of protein kinase A) each inhibited the stimulatory effects of ACh on phosphorylation of Ser19 and Ser40, respectively, while SMI addition did not augment the inhibition any further. This result validated our hypothesis that SMI may regulate the phosphorylation of Ser19 and Ser40 to influence TH activity.

Shenmai injection (SMI) has been used extensively in clinical applications to treat cardiovascular diseases. Since excessive secretion of CA plays an important role in cardiovascular diseases, it is worth investigating the effect of SMI on the secretory CA system of adrenal medullary cells. CA neurotransmitters such as dopamine (DA), epinephrine (E), and norepinephrine (NE) have vital physiological roles. NE and *E* act as central nervous system stimulants resulting in faster heart rate, enhanced myocardial contraction, increased cardiac output, elevated blood pressure, reduced splanchnic blood flow, and increased adipose decomposition and oxidation. Stress is one of the reasons for the increased secretion of calcium synthetic hormones, and chronic stress can trigger abnormal immune functions, high blood pressure, coronary heart disease, and atherosclerosis. Therefore, controlling the synthesis and secretion of CA through drug interventions has immense therapeutic potential. Ca^2+^ influx is the essential trigger for CA synthesis and secretion, and nicotinic acetylcholine receptor (nAChR) ion channels play an important role in Ca^2+^ influx. *α*3 and *α*4*β*2 as *α*-subunit sites of nAChR ion channels affect its activation. In our study, we found that SMI reduced CA synthesis and secretion induced by ACh, and cotreatment with SMI and the ACh receptor *α*-subunit inhibitors, HEX(*α*3) or Dh*β*E(*α*4*β*2), did not produce any further inhibition, indicating that SMI may reduce CA synthesis and secretion by inhibiting *α*3 and *α*4*β*2 subunit sites to suppress AChR ion channels. As a rate-limiting enzyme in the synthesis of CA, the activity of TH is often tested. TH exerts its effect by phosphorylation at Ser19 with CaM kinase II and Ser40 with PKA. We found out that SMI suppressed ACh-induced TH activity by inhibiting the phosphorylation of TH at Ser19 and Ser40. KN-93 and H89, the antagonists of CaM kinase II and PKA, respectively, inhibited the ACh-induced phosphorylation at Ser19 and Ser40, and the addition of SMI did not augment the inhibitory effect. These results indicate that SMI inhibits CA secretion by blocking TH activity at Ser19 and Ser40 sites.

The present findings would support the idea that SMI suppresses the CA secretion and synthesis by inhibiting AChR ion channels and TH activity. SMI may also delay the development of cardiovascular disease through the modulation of the sympathetic-adrenal medullary system, primarily by inhibiting the secretion of neurotransmitters at the sympathetic terminals. To confirm this possibility, further in vivo and in vitro studies will be required in near future.

## Data Availability

The data used to support the findings of this study are available from the corresponding author upon request.

## Abbreviations

Ach: Acetylcholine
CA: Catecholamines
CaM kinase II: Ca^2+^/calmodulin-dependent protein kinase II
DA: Dopamine
DH*β*E: Dihydro-*β*-erythroidine hydrobromide
DOPA: Dihydroxyphenylalanine
E: Epinephrine
ERK: Extracellular signal-regulated protein kinase
HEX: Hexamethonium bromide
KRP: Oxygenated Krebs-Ringer phosphate
MLA: Methyllycaconitine citrate
NE: Norepinephrine
PCA: Perchloric acid
PKA: cAMP-dependent protein kinase A
SMI: Shenmai injections
TH: Tyrosine hydroxylase
Ver: Veratridine.
